# The Rural Household Production of Health Approach: Applying Lessons from Zambia to Rural Cancer Disparities in the U.S.

**DOI:** 10.3390/ijerph19020974

**Published:** 2022-01-15

**Authors:** Mutale Chileshe, Emma Nelson Bunkley, Jean Hunleth

**Affiliations:** 1 Behavioural Science Unit, Clinical Sciences Department, Michael Chilufya Sata School of Medicine, Copperbelt University, Ndola P.O. Box 21692, Zambia; 2 Department of Surgery, Division of Public Health Sciences, Washington University School of Medicine, St. Louis, MO 63110, USA; bunkley@wustl.edu

**Keywords:** household, gender, generation, therapy management, distance, hospital, caregiving

## Abstract

The recent focus on rural–urban cancer disparities in the United States (U.S.) requires a comprehensive understanding of the processes and relations that influence cancer care seeking and decision making. This is of particular importance for Black, Latino, and Native populations living in rural areas in the U.S., who remain marginalized in health care spaces. In this article, we describe the household production of health approach (HHPH) as a contextually-sensitive approach to examining health care seeking and treatment decisions and actions. The HHPH approach is based on several decades of research and grounded in anthropological theory on the household, gender, and therapy management. This approach directs analytical attention to how time, money, and social resources are secured and allocated within the household, sometimes in highly unequal ways that reflect and refract broader social structures. To demonstrate the benefits of such an approach to the study of cancer in rural populations in the U.S., we take lessons from our extensive HHPH research in Zambia. Using a case study of a rural household, in which household members had to seek care in a distant urban hospital, we map out what we call a *rural HHPH approach* to bring into focus the relations, negotiations, and interactions that are central to individual and familial health care seeking behaviors and clinical treatment particular to rural regions. Our aim is to show how such an approach might offer alternative interpretations of existing rural cancer research in the U.S. and also present new avenues for questions and for developing interventions that are more sensitive to people’s realities.

## 1. Introduction

Just before the start of the rainy season in 2017, brothers Paul and Makunka and their mother, Theresa, should have been preparing their land in Luapula Province, Zambia for planting. Instead, the three were traveling to a hospital located in the city of Ndola, approximately 300 km from the family’s village. Makunka, who was 30 years old, was suffering from a disease that none of the local providers or healers he saw could resolve. The family’s only remaining option was to take him to an urban hospital that provided specialty care. The brothers and their mother had never visited Ndola nor did they have family or friends there. However, the three would find their way, first by travelling on foot, and then by using a series of buses. Once in Ndola, they would reside in the hospital for six weeks. It was at the hospital that Mutale Chileshe (first author) came to know Theresa and Paul while she was carrying out the Bedsiders’ Welfare at Ndola Teaching Hospital study with medical student, Imukusi Mutanekelwa, from 2017 to 2019. Mutale learned from them that Makunka’s wife and three children along with Paul’s wife and children had remained in Luapula to do the work of cultivating, despite great worry over Makunka’s health. She also learned of their concerns that their wives would be unable to produce the amount of food required by the family for the coming year without the additional labor that they typically provided.

The above case, despite being from Zambia, touches on issues that run throughout rural cancer disparities research in the U.S. including distance and transportation to specialty care [1,2,3], late diagnosis and treatment [2,4], financial strain [2,5], family caregiving [6,7], and time spent in or adjacent to an urban hospital (e.g., in Hope Lodge or other lodging for cancer patients and caregivers) [8]. These issues are becoming even more pressing in the U.S. as rural hospital closures reach record highs [9,10]. In a 2017 blog post, Robert Croyle, the director of the National Cancer Institute, called for innovative approaches to rural cancer disparities research, suggesting that U.S. researchers have made only modest advances in rural cancer control over the past two decades [11].

In this article, we suggest that U.S. researchers committed to addressing rural cancer disparities can learn methodological lessons from researchers and research in the Global South. We write this as anthropologists working in medical schools that are located in cities surrounded by rural regions. Given this positionality, we engage often (in our studies and university work) with rural patients seeking care in our university hospital systems. Mutale Chileshe, the first author, was a Zambian anthropologist, trained in South Africa, who carried out research in rural Zambian households and also in the hospital in Ndola where Makunka was admitted [12,13,14,15,16]. Jean Hunleth and Emma Bunkley, both Americans, have also carried out multiple household- and hospital-based studies in Zambia and elsewhere in sub-Saharan Africa (see, for example, [17,18,19,20,21,22]). Jean is also an investigator on a number of NCI-funded efforts to reduce rural cancer disparities in her U.S. NCI-designated cancer center’s catchment area. Our research expertise as well as our anthropological training have led us to see the HHPH approach as necessary to efforts to address inequalities in U.S. cancer prevention and treatment in ways that are sensitive to context, and that do not reinforce and exacerbate inequalities. The idea for this paper was born during Mutale’s visit to the U.S. in 2018, when she was able to observe rural U.S. health disparities and draw connections to her own research in Zambia. The aim of this paper is to demonstrate the importance of using a rural HHPH approach to research in rural cancer care both in the U.S. and globally. 

## 2. Toward a Rural HHPH Approach to Hospital Care

The household production of health (HHPH) approach offers a means for researchers to examine health and illness using the household as the unit of analysis, as opposed to the more narrow focus at the individual level, the doctor–patient relationship, or the caregiver–patient dyad [23,24,25]. An HHPH approach, according to Agbo et al., “situate[s] health care within the full range of activities undertaken to achieve well-being for the household as a unit” [25]. To understand the “full range of activities” and their implications for rural health, it is helpful to break this definition of HHPH into three theoretical strands. First, the approach centers the household and draws from an extensive anthropological scholarship on what households are and are not [26,27]. In an HHPH approach, households are not defined by structure or typology. Such a view would erase the dynamism and fluidity of social life and the household through the life course. Rather, an HHPH approach defines households as sites of interaction and economic exchange. Given that households are made up of members who have varying skills, information, access to resources, and, relatedly, power, they are also sites of negotiation, collaboration, and conflict [26,27,28]. Additionally, while this definition often situates interactions of people in close proximity to one another, households can be dispersed as people move in and out (e.g., for school, work, or to the hospital). Second, a focus on interactions compels an analysis of gender and generational relations in households, and offers insight into how cultural, political, and economic processes play out in and are changed through household interactions during illness (e.g., women taking primary roles as caregivers in and across households) [29]. Finally, an HHPH approach calls attention to the social basis of healing. Anthropologist John Janzen coined the term therapy management group as a corrective to the individualistic focus of health research, in which patients and doctors have been assumed to be the sole decision makers [30,31]. Janzen showed that a range of people—in households, families, and communities—become involved in quests for therapy through offering resources and influencing diagnostic and treatment decisions, and that these people need to be taken into account in health care research (see also [32,33,34,35]). 

All three theoretical strands direct analytical attention to how resources (e.g., time, money, legal, and cultural) are secured and allocated, sometimes in highly unequal ways [26,27,28]. A focus on resources in the HHPH approach allows us to locate the importance of households, as anthropologist Jane Guyer wrote, “in the wider systems to which they now belong and to theorize the dynamics of change” [27]. Such a view makes the study of households critical to understanding how macro-level forces produce health inequalities that impact individual patients and caregivers and, also, reverberate beyond the individual patient or patient–caregiver dyad. Below, we focus in on the case of Makunka to map out a rural HHPH focus through the three theoretical lenses that make up the approach. 

For rural residents in Zambia as well as in the U.S., household members of persons who are sick are likely to participate in, for example, negotiating care at a distance, organizing transportation with often limited options, finding someone to accompany the person in need of care (if possible), taking time away from jobs and other responsibilities that may not offer such flexibility, navigating complex urban centers, and dealing with a confusing health insurance system (in the U.S.) or no health insurance at all (as has been the case for many rural Zambians). These landscapes of health care in both Zambia and the U.S. have fostered a sense of improvisation, making do, and band-aid care [36], as becomes evident in our narrative account of the experiences of Makunka and his household. Makunka, Paul, and Theresa were part of a study Mutale carried out on the HHPH at a hospital in Ndola. Her methods were ethnographic, including participant observation and informal interviewing. (This study also included a quantitative survey which we do not present here. The quantitative survey was carried out by Imukusi Mutanekelwa. While we do not have the space to present it here, such quantitative work has long been a feature of ethnographic research in sub-Saharan Africa.) While we examine this single case in depth, our writing is heavily informed by findings from multiple other households participating in our studies reaching back to the early 2000s (for publications on our methods, see [14,15,37,38]).

### 2.1. Rural Households in Urban Hospitals

In Zambia, 100 percent of the tertiary referral, or specialist hospitals, are situated in urban centers (of Lusaka, Ndola, Kitwe, and Livingston), despite 56 percent of the population residing in rural regions. Thus, Theresa and Paul were among the many Zambians who accompany a loved one to the city for hospital care. In Zambian hospitals, kin or kin-like caregivers must stay with patients around the clock. They feed, bathe, watch over the sick person, and much more. They do so out of love or familial duty, and because hospital nursing staff are overburdened. Like many rural residents in our studies, Theresa, Paul, and Makunka brought with them only a small amount of money and the clothes on their backs for their relocation to the hospital grounds. 

The family’s distance from home distinguished them from hospital-goers living nearby. Their stay, like those of other rural patients, would be longer, because they did not have the means to go back and forth for diagnostic testing and treatment, nor could Makunka’s ailing body take the strain of back and forth travel. Not only that, the family was physically cut off from the members of their household who remained at home for the duration of their stay. Patients and caregivers living closer to the hospital could receive food, money, and other necessities (e.g., cooking pots and dishes) to sustain the person giving care. If other household or family members were available, they could “swap” caregivers to allow the primary caregiver to go home for a break or to carry out their other household duties. In contrast, rural householders remain at the hospital, and were limited to the resources they brought along in addition to what they were provided by hospital staff and humanitarian and church groups. 

Our research in a different hospital in Ndola during the COVID-19 pandemic highlighted this critical difference between rural and urban hospital goers [39]. COVID-19 pandemic restrictions at their strictest meant that no family caregivers, rural or urban, were able to swap out or to leave the hospital easily, for fear of community infection. This effectively collapsed the rural–urban divide, creating conditions for all like the ones that Theresa and Paul endured. There was, however, one exception: visiting hours were held at the gate, during which visitors passed through food, money, and other items to caregivers on the inside. This “giving care at the gate”, however, was only viable for people coming from close by. Such restrictions substantially strained family caregivers on the inside and put more demands on the hospital staff to make up for the absence (an absence hospital staff already experienced with rural patients, such as Makunka). 

On their own at the hospital, Theresa and Paul set up a makeshift household for themselves. They slept, ate, bathed, and washed at the hospital. They gave care to Makunka on the ward and sought resources, such as food and money, to support their stay both on and off the ward. Hospital rules, even pre-COVID-19, only permitted one person to be on the ward at a time outside of visiting hours, and so Theresa and Paul traded off, with one sitting on a metal chair beside Makunka’s hospital bed to feed and comfort him and to watch for signs of improvement or worsening of his condition. The other would rest on a bench or the lawn outside of the hospital. How they arranged their household in the hospital had much to do with gender and generational roles. 

### 2.2. Gender and Generation in Rural HHPH

Gender and generation are at the heart of decisions about who goes to the hospital and who stays home, and then, who does what during hospital stays [29]. The gendered and generational aspects of Makunka’s quest for therapy are stark. Theresa, as Makunka’s mother, expected and was expected to accompany her son to the hospital and nurse him back to health, in fitting with the belief that motherly love could help him return to health [19]. Further, with her children grown, Theresa did not have the childcare responsibilities that kept Makunka’s wife (who had three young children at home) from accompanying her husband. If the family had been from Ndola, it is likely that only Theresa would have accompanied Makunka, and that Theresa and Makunka’s wife may have swapped places during his stay. Yet, due to their distance, Paul also came.

Paul’s responsibility, as a younger man and Theresa’s son, was to support his mother to navigate the unfamiliar urban environment and support her nursing work in the hospital. With the family’s three primary breadwinners in the hospital (Makunka, Theresa, and Paul), Paul needed to find ways to earn money while in Ndola—money to be used to support Makunka’s medical costs, to pay for food for Paul and Theresa while caring for Makunka, and to send to the brothers’ wives in Luapala who needed to feed themselves and the children, maintain the home, and harvest as best they could. Paul set out each day from the hospital in search of day labor in Ndola. Each day, he returned unsuccessful and empty-handed, feeling that he had failed his mother, Makunka, and the rest of the family. 

The stay wore on Paul and Theresa, and their hunger and exhaustion increased. Mutale noticed that Theresa and Paul always looked exhausted and stressed when she met with them. Yet, she also witnessed them concealing their physical and emotional suffering from each other and, most importantly, from Makunka. We have shown elsewhere the importance of conveying positive sentiments as a form of care, meant to nurture patients and to demonstrate to others that the caregiver is fulfilling their social responsibilities [14,18,19]. However, it was clear that the lengthy hospitalization was affecting both Paul’s and Theresa’s own health.

Makunka’s and Paul’s wives, though not present at the hospital, were likewise key participants in the household response to Makunka’s illness. They played a critical role in mitigating the effects of Makunka’s illness by ensuring that the primary household(s) did not collapse under the strain of this emotionally and economically trying time. Their children, too, likely played a part. In our 2018 survey of 228 caregivers in the same hospital where Makunka was admitted, 23% told us that they left children in charge of their households. Even very young children, as we have shown in different studies in Zambia, play substantial supportive roles in maintaining households during hospitalization [18,19,20]. Take 10-year-old Steven, who participated in Jean’s *Children as Caregivers* study, for example. Steven’s father had Kaposi’s sarcoma, for which he was hospitalized frequently. Steven cooked and cleaned for his household, and he sold goods from home to keep up his mother’s business while she cared for his father in the hospital (for more on Steven, see [18,19,20]). In Makunka’s case, the older children likely cared for their younger siblings and would also have helped with farming. 

Lastly, there is a gendered aspect to Makunka’s case that is best seen when compared to other, similar cases but in which the patient is a woman rather than a man. During that same study of family caregiving, Mutale observed a young woman who was hospitalized at approximately the same time as Makunka and who arrived to the hospital after a journey similar in length and duration to Makunka’s. Unlike Makunka, the woman was accompanied only by her nursing infant. As the woman became sicker during hospitalization, hospital staff made the difficult decision to place the baby in a local orphanage for interim care. Our household-based studies reveal the additional challenges that rural and peri-urban women face when coming to and then staying in the hospital. These include unwavering (even in the face of illness) responsibilities to provide childcare and labor at home, along with starkly gendered assumptions about who should perform caregiving. We also found that women endure much higher incidence of blame and shame for their own illnesses than do men, including Makunka. Women, more likely to be without their mothers and brothers when hospitalized, relied more heavily on support offered by groups and individuals outside of the household, such as church groups, humanitarian organizations, and the goodwill of clinicians and families of other patients, and they more often needed social services that were available, though often insufficient. 

### 2.3. Therapy Management across Rural and Urban Locales

When someone migrates for hospitalization, the people involved in managing their diagnostic and treatment decisions shift. Makunka’s quest for therapy did not begin at the hospital in Ndola but closer to home, where he saw a range of healers and clinicians. Moving to Ndola meant that he had a new clinical care team. Additionally, it also meant that people who had been an intimate part of his therapy group—his wife and his children, as well as other kin and community members who had become involved—were now 300 km away. While Theresa and Paul were always influential in making decisions about Makunka’s care, especially after he became severely ill, they no longer had the in-person support of their wives and others in their village, with whom they discussed care decisions or could ask questions. Cell phones bridge the distance, but only to a degree, given the challenges with cell coverage and the expense of calls, as well as the inability for those on the other end of the calls to see for themselves a patient’s or caregiver’s condition before offering advice. 

Strangers physically proximate to Makunka’s bedside became part of his evolving therapy management group. Theresa and Paul turned to the caregivers of other patients and to Mutale (first author) for advice as they made decisions and when they were unsure of what diagnostic testing or treatment to advocate for. Among Theresa and Paul’s concerns, they still did not know from what Makunka suffered. They had not talked to the doctor because they were told to leave the ward during doctors’ rounds—a common practice that clinicians identify as important for maintaining patient privacy, preventing overcrowding and noisiness, and avoiding potential conflicts with caregivers. Theresa and Paul, as well as other bedsiders, expressed frustration with this practice. However, even when they had the opportunity to speak with clinicians, they struggled. They spoke the same language as the doctors and other staff at the hospital, but their dialect, along with their dress, comportment, and education level communicated rurality. This rurality shaped the way clinical and hospital staff viewed Paul and Theresa; it threatened their belonging in the hospital, and questioning clinical authority figures made them feel uncomfortable. With little professional input, they turned to other caregivers on the ward to make decisions about Makunka’s care. Even so, without the social and financial resources, they could not affect changes in Makunka’s diagnosis and treatment.

Makunka died six weeks after his arrival to the hospital. Following his death, Paul and Theresa no longer had decisions to make about diagnosis or treatment. However, as his body laid in the hospital’s morgue, they were faced with new questions about how to mourn Makunka and where and how to lay him to rest. Theresa and Paul had no money left with which to bury Makunka in Ndola or to transport his body back for burial in the village. The “worst part of it”, Theresa said, was that the family did not have a private place to mourn in the hospital. Seeing Theresa’s suffering, another family caregiver in the hospital who lived in Ndola offered Theresa and Paul her house to “help them cry in peace and with dignity”. Hospital staff who witnessed these struggles leveraged resources through the social work office and also donated personal money, ultimately enabling Theresa and Paul to bury Makunka in Ndola and then return home. While better than nothing, it is important to note that the decision to bury Makunka in Ndola was a result of financial necessity, rather than agreed upon by members of the family usually in the position to make such decisions. The Ndola burial excluded from funerary rituals most who mourned Makunka, including his wife, children, and friends and neighbors at home in Luapala. It also displaced Makunka’s body, by 300 km, from those of his ancestors. Additionally, Paul and Theresa’s return home without Makunka’s body for burial was likely to raise doubts about the therapy management decisions they made while in Ndola and prompt critical conversations about what they could have done better. Women caregivers, like Theresa, we have found in our prior work, are especially vulnerable to blame when the person in their care dies [19].

## 3. Toward an HHPH Approach to U.S. Rural Cancer Disparities Research

An HHPH approach offers a needed lens for researchers to examine patient health care decisions and outcomes in relation to a multiplicity of people and within larger political and economic structures and influences. A rural HHPH approach, as we define it in this article, centers rural households and relations across rural and urban spaces, including in the hospital. We have drawn on Makunka, Paul, and Theresa’s experience to demonstrate the HHPH approach, by first demonstrating how households become dispersed, with implications on resource availability, including modes of production and employment. Then, we focused more specifically on gender and generational relations as they shape and are changed by treatment seeking. Finally, we identified the shifts in who influences therapy management as rural households move away from their social networks and, importantly, must confront different modes of communication, new treatment and diagnostic decisions, and discrimination and bias related to being rural. We show here how rurality, gender, and poverty intersect. 

A rural HHPH approach in the U.S. requires also acknowledging both the existence of Black, Latino, and Native people in rural areas, and how the institutionalization of racism has affected where Black, Latino, and Native people live and work, and if and how they are able to seek health care [40]. Further, as Sangaramoorthy has shown in relation to immigrants in the U.S., the “precarity that characterizes rural health systems is intensified for growing communities of immigrants, who must constantly contend with the broader logics of exclusion that are at the heart of health-care and immigration reform, while also seeking care” [36]. The implications of historical and current day policies on households and on health are evident in the rural cancer research in the U.S., and are particularly pressing for Black, Latino, and Native residents of rural areas [2,41]. Distance to specialists and the shuttering of rural hospitals has impacted rural care seeking. On average, rural breast cancer patients must travel nearly three times as far and colorectal patients almost eight times as far for care as their urban counterparts [42,43]. When mapping distances to oncology specialists in the U.S., Hung and colleagues identified substantial distances to specialists for rural and low-income communities, and found that distances were greater for communities with higher percentages of American Indian/Alaska Native residents [43]. Zahnd et al. further showed that rural cancer survivors face greater financial problems due to cancer than their urban counterparts, and they identified the interplay of rurality and race [5]. 

A rural HHPH approach is needed to expand understandings of these U.S.-based, rural cancer disparities and what is needed to address such disparities without further exacerbating the financial and social situations of families. Such an approach would take into considerations the many household members who may be involved in hospital care, yet may also be invisible to providers because they remain at home. It acknowledges that households in the U.S. vary tremendously and can include multiple generations and care configurations. A careful, situated ethnographic approach to family and household caregiving, as Fayana Richards suggests in her study of Black grandmothers caring for their grandchildren in Detroit, can upend racist stereotypes, for example, about Black women and Black American families [44]. The HHPH approach also aims to correct inaccurate assumptions that researchers and policymakers hold about what co-residence and the distribution of care might look like, a point Henderson makes in his study of American Indian family caregiving in rural Oklahoma [45]. An HHPH approach also offers insight into the gender and generational inequalities in care that continue to be exacerbated in the U.S.-based health care system that relies on women, and especially women of color, to take up much care work [44]. An HHPH approach can bring these important issues related to structural inequalities to the fore by addressing topics of interest to rural disparities researchers, as we have shown in this article (e.g., distance, transportation, financial strain, and communication). It also complements current work being carried out in the U.S. that has strong backing from funding agencies, such as the National Institutes of Health. For example, an increasing number of rural cancer disparities researchers have turned to multi-level interventions to address cancer control at the individual, household, clinic/hospital, and community levels [46]. An HHPH approach fits such a model by acknowledging the connections and relations among these levels, including during times of dramatic change, such as during the COVID-19 pandemic, which has changed the resources available within households and household members’ mobility. 

Alewine et al. recently wrote that, during the COVID-19 pandemic, oncology providers are “fighting cancer with half the team” [47]. By half the team, they mean that family caregivers were not allowed into hospitals with patients due to COVID-19 pandemic restrictions. The article focuses on a case of a man coming from 250 miles away, who was previously accompanied by his wife and the care loss because of her absence. The HHPH approach complements this increasing attention to family cancer caregivers that has made important contributions into identifying the interdependence between patients and caregivers, and the gendered dimensions of caregiving [48,49,50]. By widening the lens to the household, the HHPH approach can examine a range of influences on dyadic care, and the roles and outcomes of caregivers and patients. 

Our addition of rural to the HHPH approach is also critical for understanding what it takes for rural patients to get to hospitals. Such an approach moves beyond a focus on distance and transportation to a focus on how resources are secured and allocated to support some householders to go and others to stay. A focus on households can reveal the amount of labor that goes into keeping up the household during hospitalization, and how the temporal and financial costs of cancer diversely affect householders in different scenarios. By making a range of activities and persons visible within cancer therapy management, the HHPH approach can also show providers what and who it takes to support cancer patients, and reveal provider assumptions about caregiving (e.g., that children do not give care). This is necessary so as not to perpetuate and reinforce inequalities, not just in cancer diagnosis, treatment, and survivorship, but also in the many and myriad ways cancer impacts households and communities, a needed addition to the burgeoning research showing the financial strain caused by cancer. 

## 4. Conclusions

Despite 56 percent of the Zambian population residing in rural areas of the country, 100 percent of the tertiary referral, or specialist hospitals are situated in urban centers (of Lusaka, Ndola, Kitwe, Livingston). The United States—though distant in many ways from the Zambian case we present—also faces unevenness in the distribution of hospitals, and a continuing disinvestment in rural health care. For this reason, we suggest that researchers working in U.S. rural health disparities might learn from our on-going research that uses a rural HHPH approach in Zambia. By using a rural HHPH approach, U.S.-based researchers can learn the ways people seek treatment, balance finances that may already be strained, live within multi-morbid families, and access social and economic resources. Rather than focusing on individual behavior and treatment, the HPPH approach insists that researchers and clinical providers situate individuals within their household structure to truly understand decisions being made and the best approach to treatment. When designing and implement interventions, whether behavioral, systems, or policy, a more situated view of how households organize care is needed. Centering the household in both research and implementation will help to identify how longstanding exclusionary policies and practices play out in health seeking and treatment and will improve the lives and outcomes of cancer patients, their caregivers, and their households.

## 5. Post Script

Dr. Chileshe, the first author of this article, died in February 2021. To find out more about her career and the tremendous impact she had on medical training and hospital care in Zambia, see Hunleth and Willis 2021 and Asante et al. 2021. We also encourage readers to read her articles, which, just as her intention in this article, offer lessons for researchers around the world, while remaining grounded in the Zambian case. See especially Chileshe 2016, where she discusses researchers’ emotions during research. This article is especially useful for training students and research assistants new to qualitative research addressing health inequities. See also Chileshe 2021, on training medical students in participatory anthropology.

## Data Availability

Due to confidentiality issues, we are not making the data available.

## References

[1] Zahnd W.E., Mueller G.S., Fogleman A.J., Jenkins W.D. (2016). Intrastate Variations in Rural Cancer Risk and Incidence: An Illinois Case Study. J. Public Health Manag. Pract..

[2] Kruse-Diehr A.J., Lewis-Thames M.W., Wiedenman E., James A., Chambers L. (2021). Perspectives of cancer prevention and control resources from stakeholders in rural southern Illinois. J. Rural Health.

[3] Zahnd W.E., James A.S., Jenkins W.D., Izadi S.R., Fogleman A.J., Steward D.E., Colditz G.A., Brard L. (2018). Rural-Urban Differences in Cancer Incidence and Trends in the United States. Cancer Epidemiol. Biomark. Prev..

[4] Chandak A., Nayar P., Lin G. (2019). Rural-Urban Disparities in Access to Breast Cancer Screening: A Spatial Clustering Analysis. J. Rural Health.

[5] Zahnd W.E., Davis M.M., Rotter J.S., Vanderpool R.C., Perry C.K., Shannon J., Ko L.K., Wheeler S.B., Odahowski C.L., Farris P.E. (2019). Rural-urban differences in financial burden among cancer survivors: An analysis of a nationally representative survey. Support. Care Cancer.

[6] Kent E.E., Rowland J.H., Northouse L., Litzelman K., Chou W.Y., Shelburne N., Timura C., O’Mara A., Huss K. (2016). Caring for caregivers and patients: Research and clinical priorities for informal cancer caregiving. Cancer.

[7] Kent E.E., Ornstein K.A., Dionne-Odom J.N. (2020). The Family Caregiving Crisis Meets an Actual Pandemic. J. Pain Symptom Manag..

[8] Daniel G., Wakefield C.E., Ryan B., Fleming C.A., Levett N., Cohn R.J. (2013). Accommodation in pediatric oncology: Parental experiences, preferences and unmet needs. Rural Remote Health.

[9] Kaufman B.G., Thomas S.R., Randolph R.K., Perry J.R., Thompson K.W., Holmes G.M., Pink G.H. (2016). The Rising Rate of Rural Hospital Closures. J. Rural Health.

[10] Wishner J., Solleveld P., Rudowitz R., Paradise J., Antonisse L. (2016). A Look at Rural Hospital Closures and Implications for Access to Care: Three Case Studies. The Kaiser Commission on Medicaid and the Uninsured. https://files.kff.org/attachment/issue-brief-a-look-at-rural-hospital-closures-and-implications-for-access-to-care.

[11] Croyle R.T., National Cancer Institute (2017). Improving cancer control in rural communities: Next steps. Cancer Current Blogs.

[12] Chileshe M. (2008). Tuberculosis, HIV, Food Security, and Poverty in Rural Zambia: An Ethnographic Account of the Southern Province.

[13] Chileshe M. (2014). Economic Shocks, Poverty and Household Food Insecurity in Urban Zambia: An Ethnographic Account of Chingola.

[14] Chileshe M. (2016). Emotional reflexivity: Fieldwork among vulnerable and poor populations. Pract. Anthropol..

[15] Chileshe M., Martinez I.L., Wiedman D.W. (2021). Participatory Anthropology for Teaching Behavioral Sciences at a Medical School in Zambia. Anthropology in Medical Education: Sustaining Engagement and Impact.

[16] Chileshe M., Bond V. (2010). Barriers and outcomes: TB patients co-infected with HIV accessing antiretroviral therapy in rural Zambia. AIDS Care.

[17] Hunleth J., Jacob R., Cole S., Bond V., James A. (2014). School holidays: Examining childhood, gender norms, and kinship in children’s shorter-term residential mobility in urban Zambia. Child. Geogr..

[18] Hunleth J. (2019). Zambian children’s imaginal caring: On fantasy, play, and imagination in an epidemic. Cult. Anthropol..

[19] Hunleth J. (2017). Children as Caregivers: The Global Fight Against Tuberculosis and HIV in Zambia.

[20] Hunleth J. (2013). Children’s roles in tuberculosis treatment regimes: Constructing childhood and kinship in urban Zambia. Med. Anthropol. Q..

[21] Hunleth J. (2013). “ARVs” as sickness and medicine: Examining children’s knowledge and experience in the HIV era in urban Zambia. AIDS Care.

[22] Bunkley E. (2021). Diagnosing diabetes, diagnosing colonialism: An ethnography of the classification and counting of a Senegalese metabolic disease. Med. Anthropol. Theory.

[23] Berman P., Kendall C., Bhattacharyya K. (1994). The household production of health: Integrating social science perspectives on micro-level health determinants. Soc. Sci. Med..

[24] Nichter M., Kendall C. (1991). Beyond child survival: Anthropology and international health in the 1990s. Med. Anthropol. Q..

[25] Agbo I.E., Johnson R.C., Sopoh G.E., Nichter M. (2019). The gendered impact of Buruli ulcer on the household production of health and social support networks: Why decentralization favors women. PLoS Negl. Trop. Dis..

[26] Hansen K.T. (1997). Keeping House in Lusaka.

[27] Guyer J. (1981). Household and Community in African Studies. Afr. Stud. Rev..

[28] Berry S. (1989). Social institutions and access to resources. Africa.

[29] Clark L. (1993). Gender and generation in poor women’s household health production experiences. Med. Anthropol. Q..

[30] Janzen J.M. (1987). Therapy Management: Concept, Reality, Process. Med. Anthropol. Q..

[31] Janzen J.M., Arkinstall W. (1978). The Quest for Therapy in Lower Zaire.

[32] Mkhwanazi N., Manderson L. (2020). Connected Lives: Families, Households, Health and Care in South Africa.

[33] Livingston J. (2005). Debility and the Moral Imagination in Botswana.

[34] Feierman S., Janzen J.M. (1992). The Social Basis of Health and Healing in Africa.

[35] Whyte S.R. (2014). Second Chances: Surviving AIDS in Uganda.

[36] Sangaramoorthy T. (2018). “Putting Band-Aids on Things That Need Stitches”: Immigration and the Landscape of Care in Rural America. Am. Anthropol..

[37] Hunleth J. (2011). Beyond on or with: Questioning Power Dynamics and Knowledge Production in “Child-Oriented” Research Methodology. Childhood.

[38] Asante C., Burack S., Chileshe M., Hunleth J. (2021). Co-producing knowledge and care in team-based fieldwork in the COVID-19 era. Anthropol. S. Afr..

[39] Hunleth J., Asante C., Burack S., Chileshe M. (2021). Care at the Gate. Anthropol. News.

[40] Benton A., Sangaramoorthy T., Kalofonos I. (2017). Temporality and Positive Living in the Age of HIV/AIDS: A Multi-Sited Ethnography. Curr. Anthr..

[41] Kruse-Diehr A.J., McDaniel J.T., Lewis-Thames M.W., James A.S., Yahaya M. (2021). Racial Residential Segregation and Colorectal Cancer Mortality in the Mississippi Delta Region. Prev. Chronic Dis..

[42] Longacre C.F., Neprash H.T., Shippee N.D., Tuttle T.M., Virnig B.A. (2020). Evaluating Travel Distance to Radiation Facilities Among Rural and Urban Breast Cancer Patients in the Medicare Population. J. Rural Health.

[43] Hung P., Deng S., Zahnd W.E., Adams S.A., Olatosi B., Crouch E.L., Eberth J.M. (2020). Geographic disparities in residential proximity to colorectal and cervical cancer care providers. Cancer.

[44] Richards F. (2021). “Stepping in the gap to make family”: Care calculation and grandparent caregiving in Detroit, Michigan. J. Anthropol. N. Am..

[45] Henderson J.N. (2009). American Indian family caregiving: Cultural context and dementia patient care. Anthropol. News.

[46] Kruse-Diehr A.J., Oliveri J.M., Vanderpool R.C., Katz M.L., Reiter P.L., Gray D.M., Pennell M.L., Young G.S., Huang B., Fickle D. (2021). Development of a multilevel intervention to increase colorectal cancer screening in Appalachia. Implement. Sci. Commun..

[47] Alewine C., Ahmad M., Shea R. (2021). Caregiver Exclusion in the Age of COVID: Fighting Cancer With Half the Team. J. Clin. Oncol..

[48] Ketcher D., Otto A.K., Vadaparampil S.T., Heyman R.E., Ellington L., Reblin M. (2021). The Psychosocial Impact of Spouse-Caregiver Chronic Health Conditions and Personal History of Cancer on Well-being in Patients With Advanced Cancer and Their Caregivers. J. Pain Symptom Manag..

[49] Ketcher D., Thompson C., Otto A.K., Reblin M., Cloyes K.G., Clayton M.F., Baucom B.R.W., Ellington L. (2021). The Me in We dyadic communication intervention is feasible and acceptable among advanced cancer patients and their family caregivers. Palliat. Med..

[50] Ketcher D., Trettevik R., Vadaparampil S.T., Heyman R.E., Ellington L., Reblin M. (2019). Caring for a spouse with advanced cancer: Similarities and differences for male and female caregivers. J. Behav. Med..

